# A High Density Consensus Map of Rye (*Secale cereale* L.) Based on DArT Markers

**DOI:** 10.1371/journal.pone.0028495

**Published:** 2011-12-06

**Authors:** Paweł Milczarski, Hanna Bolibok-Brągoszewska, Beata Myśków, Stefan Stojałowski, Katarzyna Heller-Uszyńska, Magdalena Góralska, Piotr Brągoszewski, Grzegorz Uszyński, Andrzej Kilian, Monika Rakoczy-Trojanowska

**Affiliations:** 1 Department of Plant Genetics, Breeding and Biotechnology, West-Pomeranian University of Technology, Szczecin, Poland; 2 Department of Plant Genetics, Breeding and Biotechnology, Warsaw University of Life Sciences, Warsaw, Poland; 3 Diversity Arrays Technology P/L, Canberra, Australia; 4 Laboratory of Mitochondrial Biogenesis, International Institute of Molecular and Cell Biology, Warsaw, Poland; University of Massachusetts Amherst, United States of America

## Abstract

**Background:**

Rye (*Secale cereale* L.) is an economically important crop, exhibiting unique features such as outstanding resistance to biotic and abiotic stresses and high nutrient use efficiency. This species presents a challenge to geneticists and breeders due to its large genome containing a high proportion of repetitive sequences, self incompatibility, severe inbreeding depression and tissue culture recalcitrance. The genomic resources currently available for rye are underdeveloped in comparison with other crops of similar economic importance. The aim of this study was to create a highly saturated, multilocus linkage map of rye via consensus mapping, based on Diversity Arrays Technology (DArT) markers.

**Methodology/Principal Findings:**

Recombinant inbred lines (RILs) from 5 populations (564 in total) were genotyped using DArT markers and subjected to linkage analysis using Join Map 4.0 and Multipoint Consensus 2.2 software. A consensus map was constructed using a total of 9703 segregating markers. The average chromosome map length ranged from 199.9 cM (2R) to 251.4 cM (4R) and the average map density was 1.1 cM. The integrated map comprised 4048 loci with the number of markers per chromosome ranging from 454 for 7R to 805 for 4R. In comparison with previously published studies on rye, this represents an eight-fold increase in the number of loci placed on a consensus map and a more than two-fold increase in the number of genetically mapped DArT markers.

**Conclusions/Significance:**

Through the careful choice of marker type, mapping populations and the use of software packages implementing powerful algorithms for map order optimization, we produced a valuable resource for rye and triticale genomics and breeding, which provides an excellent starting point for more in-depth studies on rye genome organization.

## Introduction

Rye (*Secale cereale* L.) is the second most important cereal in Europe, where it is cultivated on 5.8 million hectares [http://faostat.fao.org]. The species is relatively high yielding under environmental conditions in which other crops perform poorly even with low chemical inputs, such as fertilizers and pesticides, enabling ecologically and economically sound cultivation [1]. Rye also plays an important role as a source of alien genes for wheat (*Triticum aestivum* L.): the 1BL.1RS or 1AL.1RS translocation is present in hundreds of wheat cultivars [2]. In addition, rye is a donor of the R genome to triticale (X *Triticosecale* Wittmack), a synthetic wheat-rye hybrid that occupies a significant niche in European agriculture.

Several genetic maps of different rye populations have been constructed with various marker technologies, including isozymes, hybridization-based Restriction Fragment Length Polymorphism (RFLP), and a variety of PCR-based markers, such as Amplified Fragment Length Polymorphism (AFLP), Simple Sequence Repeats (SSR), Random Amplified Polymorphic DNA (RAPD), Sequence Tagged Sites (STS), and Sequence Characterized Amplified Regions (SCAR)[3]–[16]. The major drawback of these maps and limitation to their practical application are the small number of markers and poor map density, the laborious and complicated nature of the technology employed, and the anonymous nature of the markers. The relationships between the maps of various populations have not been well studied. Börner and Korzun [17] summarized the status of consensus molecular mapping in rye, but integrated maps have only been presented by Stojałowski et al. [18] for the 6R chromosome and by Gustafson et al. [19] for all seven chromosomes from five populations.

Overall, the progress in genetic linkage mapping of rye has lagged behind that of other cereals. This is due to several factors including (i) an enormous genome (1C = 7917 Mbp [20]) containing a large proportion of repetitive sequences, (ii) inbreeding depression which has hampered the development of large recombinant inbred line (RIL) populations, (iii) tissue culture recalcitrance which has prohibited the efficient generation of doubled haploid (DH) populations, and (iv) the absence of a high throughput genotyping technology producing numerous polymorphic markers.

Diversity Arrays Technology (DArT) is a microarray-based genotyping method in which whole-genome fingerprints are generated by scoring the presence or absence of genomic DNA loci [21]. DArT alleviates a number of the limitations of gel-based marker technologies by enabling the simultaneous scoring of several thousand loci in a single assay in a largely automatic, highly reproducible and cost-effective manner. Moreover, unlike the majority of the existing Single Nucleotide Polymorphism (SNP) genotyping platforms, DArT does not rely on DNA sequence information [21]–[22]. DArT markers have been developed and successfully applied to genetic analyses in a number of plant species [22], including wheat [23], barley [24] rye [25], and triticale [26]–[28].

The development of an 11,520-clone DArT array for rye enabled the creation of a high-density map of the rye cross L318 × L9 containing over 1000 loci [25]. With an average density of one marker every 2.7 centiMorgans (cM), this was the most saturated genetic map of the rye genome, containing exclusively transferable markers, and the first created using a microarray based technology. This study also revealed several thousand DArT markers differentiating the parents of other crosses used in rye genetic mapping. The availability of numerous markers segregating in multiple populations is a prerequisite for the construction of integrated consensus linkage maps, which are invaluable for obtaining more complete genome coverage and a better understanding of its structure, precise comparison of quantitative trait loci (QTL) locations, and also anchoring of a physical map.

Our aim in this study was to create a saturated consensus linkage map of rye based on DArT marker data from five RIL mapping populations: L318 × L9, 541 × Ot1-3, Ds2 × RXL10, S120 × S76 and 541 × 2020. We constructed an integrated map containing 4048 loci, which represents an eight-fold increase in the number of loci placed on a consensus map and a more than two-fold increase in the number of genetically mapped DArT markers compared with previously published studies on rye [19], [25].

## Materials and Methods

### Mapping populations

Five RIL mapping populations, originating from 9 parental lines, were used in this study. Information on the origin and pedigree of the parental inbred lines is given in Table 1. Subsets of the parental lines were previously included in studies of rye genetic diversity that indicated a high level of polymorphism between the parents of individual populations [25], [29]–[31]. The parental lines also exhibited contrasting phenotypes with respect to several traits (Table 2). The L318 × L9 (H) population was used previously by Bolibok–Brągoszewska et al. [25] for the construction of a high density DArT-based map, which also included several SSR anchor markers. The RIL mapping population L was developed from the F_2_ progeny of the cross Ds2 × RXL10 used by Devos et al. [3] for the construction of a linkage map of rye with RFLP markers, which was later saturated with PCR-based markers [11]. This mapping population is considered a reference for linkage mapping studies in rye. F_2_-based maps were also created using PCR-based markers for the crosses 541 × OT1-3 (K) and S120 × S76 (M) [14], [32]. Additionally, for the crosses K and L, low-density maps based on RILs were constructed using selected markers from the respective F_2_ maps [33]. No existing linkage data was available for the population 541 × 2020 LM (S).

**Table 1:** Origin and pedigree for parental lines of RIL populations.

Inbred line	Origin^a^	Pedigree
541	KGHiBR Szczecin	KaH9× [(MS69-8-1xSmolickie)F_2_MS×KaH]F_1_MP
2020 LM	IHAR	Unknown
Ds2	KGHiBR Szczecin	*S. dighoricum* × Smolickie
L318	KGHiBR Warsaw	Pancerne
L9	KGHiBR Warsaw	Dankowskie Selekcyjne
Ot1-3	KGHiBR Szczecin	Otello
RXL10	KGHiBR Szczecin	Zeelandzkie
S120	DANKO	LG3 × Szk.10
S76	DANKO	LG3 × Amilo

a Origin: DANKO – DANKO Plant Breeding Ltd, Choryn, Poland; IHAR – Plant Breeding and Acclimatization Institute – National Research Institute, Radzikow, Poland; KGHiBR Szczecin – Department of Plant Genetics, Breeding and Biotechnology, West-Pomeranian University of Technology, Szczecin, Poland; KGHiBR Warsaw – Departament of Plant Genetics, Breeding and Biotechnology, Warsaw University of Life Sciences, Warsaw, Poland.

**Table 2:** Characteristics of RIL mapping populations.

Population	Code	Size	Traits segregating	Reference
L318 × L9	H	82	yield components	[13]
			heading date	
			grain characteristics	
			tissue culture response	
541 × Ot1-3	K	144	yield components	[33]
			heading date	
			grain characteristics	
			restoration of male fertility in CMS-C	
			α-amylase activity	
			preharvest sprouting	
Ds2 × RXL10	L	103	yield components	[33]
			heading date	
			grain characteristics	
			α-amylase activity	
			preharvest sprouting	
			plant height	
S120 × S76	M	143	heading date	[59]
			α-amylase activity	
			preharvest sprouting	
541 × 2020 LM	S	92	heading date	[60]
			plant height	
			restoration of male fertility in CMS-C	

### Genotyping

#### DNA extraction

Genomic DNA was extracted from around 100 mg of tissue from 2-week-old leaves using a DNeasy Plant Mini Kit (Qiagen) for populations K and L, and a GenElute^™^ Plant Genomic DNA Miniprep Kit (Sigma) for populations M and S.

#### DArT markers

DArT genotyping of RILs was performed as described previously by Bolibok-Brągoszewska et al. [25]. Genomic representations of individual RILs were prepared using the complexity reduction method involving digestion with endonucleases *Pst*I and *Taq*I, labeled with Cy3 or Cy5 by random priming and hybridized with the rye genotyping array 2.0, consisting of 11,520 probes and described in detail elsewhere [25]. Each slide was hybridized with two separate representations, labeled with Cy3 and Cy5, respectively. Images of microarrays were acquired using a confocal laser scanner (Tecan LS300, Grödig, Salzburg, Austria). Polymorphic markers were identified and scored with dedicated software (DArTsoft version 7.3, Diversity Arrays Technology P/L, Yarralumla, Australia, http://www.diversityarrays.com/software.html). The quality of the DArT markers was evaluated based on two parameters computed by DArTsoft: (i) the Q value (an ANOVA-based quality parameter indicating how well two clusters – present “1” vs. absent “0” – are separated in the set of genomic representations, with high Q values denoting reliable markers), and (ii) the call rate (the percentage of DNA samples with defined ‘0’ or ‘1’allele calls). Only markers with Q > 80% and a call rate of at least 90% were used in subsequent analyses, i.e. mapping and the calculation of a pair-wise genetic similarity (GS) matrix for the parental lines based on Jaccard’s coefficient [34] with the help of NTSYS-pc, Version 2.1. [35]. The values of GS for each possible pair of the parental lines were visualized using Circos [36].

In the case of population H, the data set used for consensus mapping and map integration contained segregations of DArT markers that were placed on a previously published map of the cross [25].

#### PCR-based markers

Several types of PCR-based markers were used to genotype: (i) SSR markers were analyzed in populations K and L according to Milczarski et al. [14], while for genotyping in populations M and S the protocol described by Stojałowski et al. [18] was used, and segregations of SSRs in population H were determined in an earlier study [25]; (ii) SCAR marker assays were performed as described by Stojałowski et al. [37]; (iii) STS marker genotyping was performed using the procedure of Milczarski et al. [14]; (iv) an Inter Simple Sequence Repeat (ISSR) marker in the population M, and (v) RAPD markers in populations K, L and M were analyzed according to Masojć et al. [9]. Information concerning the previously published PCR-based markers used for genotyping in this study is summarized in Table S1. The sequences of all other primers are available from the authors upon request.

#### Marker nomenclature

DArT marker names were automatically generated by a DArT Laboratory Information Management System with the letters *‘rPt’* added before the clone number. For all marker types, the prefix ‘*X’* was included in the name, as proposed by Schlegel and Korzun [38].

### Construction of individual maps

Individual genetic maps of the five rye RIL mapping populations were constructed using JoinMap 4.0 [39]. Prior to map construction, all marker segregations were subjected to the Chi^2^ test using the ‘locus genotype frequencies’ feature of Join Map 4.0, and severely distorted markers, deviating from the expected segregation ratio at the probability level p<0.001 (p<0.0005 in the case of population H), were excluded from further analyses. Linkage groups were separated using the independence LOD score ≥3.0. PCR-based markers with known chromosomal locations (listed in Table S1) were used to assign linkage groups to chromosomes. The order of markers within linkage groups was established with the maximum likelihood (ML) mapping algorithm and the Kosambi mapping function was used to calculate the cM values. In the process of constructing maps of individual crosses, the maps of populations H, L and K were first prepared using loci from preexisting linkage maps of these crosses to act as frameworks for saturation with DArTs. Then the information from the three newly created maps was used for assigning linkage groups to chromosomes in the remaining two maps: S and M.

### Consensus mapping

The segregation data and the marker orders established for individual populations using JoinMap 4.0 (input maps) were entered into the Multipoint Consensus 2.2 software package [40]. Assigning markers to linkage groups was repeated, this time using a recombination frequency threshold value of 0.2. Multilocus ordering combined with iterative re-sampling was performed for each data set to evaluate the stability of marker orders in the individual maps. For a correctly ordered map, the distance from a marker to its adjacent neighbor, then to the next neighbor, and so on, will grow monotonically, and a deviation from monotony indicates the presence of problematic markers. Unstable neighborhood markers were detected by the jackknife re-sampling procedure. The ‘control of monotony’ function on a hard threshold level (1.4) was used to remove problematic markers and improve the quality of the map. The general intention of the ‘control of monotony’ is to achieve maximal map stability with minimal loss of markers. Next, a consistent order (consensus order) of shared markers (i.e. markers occurring in the individual maps of at least two populations) for each linkage group was identified by the software for subsequent use in the construction of the consensus maps. In cases where two or more shared markers were co-segregating, only the first marker in such groups, named the main shared marker, was included in the consensus order. In consensus mapping, the ‘global analysis’ option was used with a heuristic algorithm ‘full frame’ for a global discrete optimization. These analyses resulted in two types of genetic map: the consensus maps of five populations and the integrated map. The consensus maps consisted of all shared markers plus unique markers (i.e. specific for an individual population), and included estimated distances between loci (in cM), which were derived from the recombination ratio distances using the Kosambi mapping function. The integrated map included shared markers and unique markers without specifying the distances between them. The consensus maps were visualized using the software MapChart [41], while the graphical presentation of the integrated map was obtained using the software Graphviz [http://www.graphviz.org].

## Results

### Construction of individual maps

**Figure 1:**
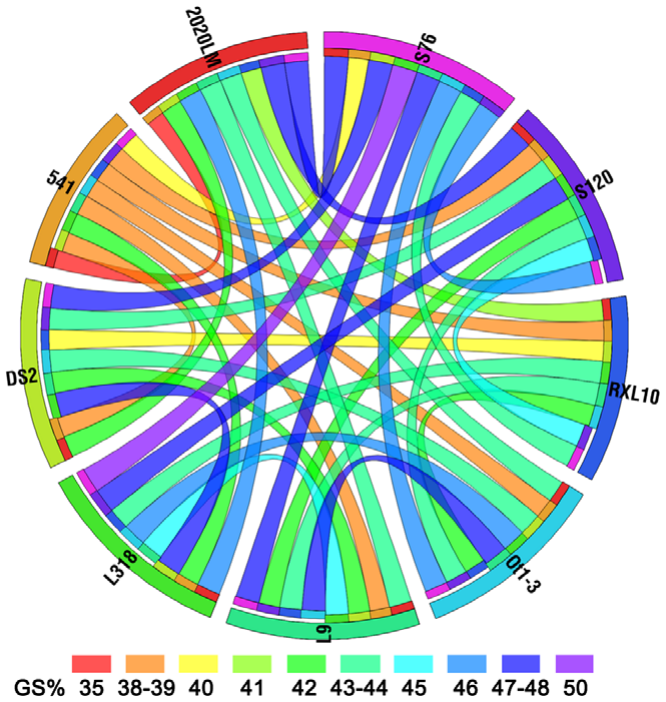
Genetic similarity of the parental lines. Genetic similarity (%) for all 36 possible line pairs is indicated by the ribbon color.

In total, 9703 marker segregations were obtained: 9563 DArT and 140 PCR-based. The DArT marker segregations for all populations are given in Table S2. The values of Jaccard’s similarity coefficient calculated based on the DArT marker scores revealed that the parental lines differed from each other to a similar extent. In pairs of the parental lines, the Jaccard’s similarity coefficient values ranged from 0.35 (S) to 0.46 (M) with an average of 0.41, while the number of segregating DArT markers in common between pairs of mapping populations ranged from 392 for H and L to 681 for K and S (Table 3). The mean value of Jaccard’s coefficient for all 36 possible genotype pairs was 0.43 and it ranged from 0.35 to 0.50 for the pair L318 and S76 (Figure 1). The number of segregations available for the construction of individual maps varied from 1689 for cross M to 2281 for cross S (Table 4), with 4403 DArT markers segregating in at least one population. Severely distorted segregation occurred in the case of 667 markers (6.9%) and these were excluded from subsequent analyses. Inspection of the linkage groups obtained using JoinMap 4.0 revealed 72 multilocus DArT markers (1.6% of markers segregating in at least one population) mapping to different chromosomes in different populations (172 segregations in total), which were then removed from the data sets. At this stage of the analyses 8303 markers were placed in linkage groups constituting the input maps, from 1352 for population M to 1942 for population S. The remaining 561 unlinked markers were not retained for consensus mapping. The numbers of markers at subsequent stages of mapping are shown in Table 4. The excluded markers are listed in Table S3.

**Table 3:** Genetic similarity coefficients for parental lines (diagonal) and number of common markers between population pairs (above the diagonal).

	H	K	L	M	S
H	0.45	445	392	472	523
K		0.38	419	417	681
L			0.40	492	474
M				0.46	543
S					0.35

**Table 4:** Summary of marker data at subsequent stages of mapping in individual populations.

	H	K	L	M	S	Total
Total number of segregations	1818	2123	1792	1689	2281	**9703**
Skewed markers (p<0.001)	46*	186	102	213	120	**667**
Markers unlinked at LOD 3.0 (JoinMap)	0	203	93	89	176	**561**
Multilocus markers	5	51	38	35	43	**172**
Markers placed in JoinMap input maps	1767	1683	1559	1352	1942	**8303**
Markers unlinked at recombination level 0.2 (MultiPoint)	73	77	83	20	73	**326**
Markers removed during control of monotony	96	141	85	14	110	**446**
Markers retained for consensus mapping	1598	1465	1391	1318	1759	**7531**

*p<0.0005.

### Consensus mapping

**Figure 2:**
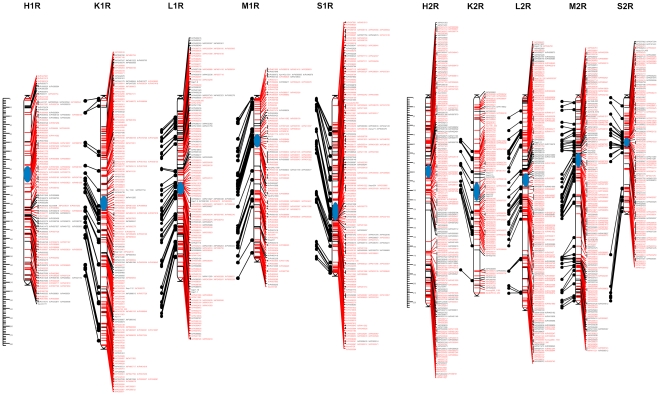
Consensus maps of chromosomes 1R and 2R. Shared and unique markers are shown in red and black, respectively. Common loci are joined by black lines. The chromosomes are oriented with the short arm at the top. The ruler shows the distance in centimorgans (cM) from the top of each chromosome. The approximate centromere locations are shown in blue.

**Figure 3:**
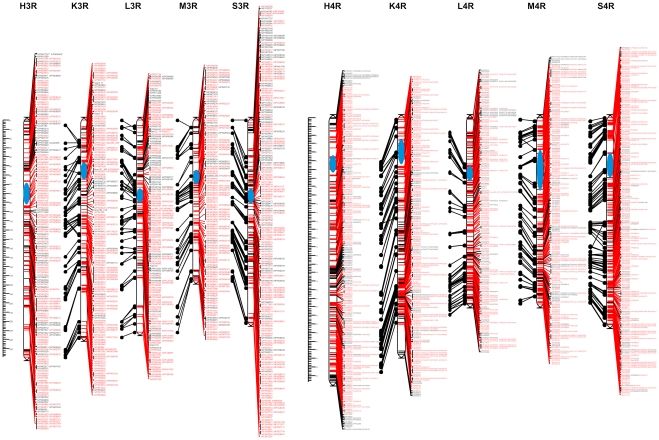
Consensus maps of chromosomes 3R and 4R. Shared and unique markers are shown in red and black, respectively. Common loci are joined by black lines. The chromosomes are oriented with the short arm at the top. The ruler shows the distance in centimorgans (cM) from the top of each chromosome. The approximate centromere locations are shown in blue.

**Figure 4:**
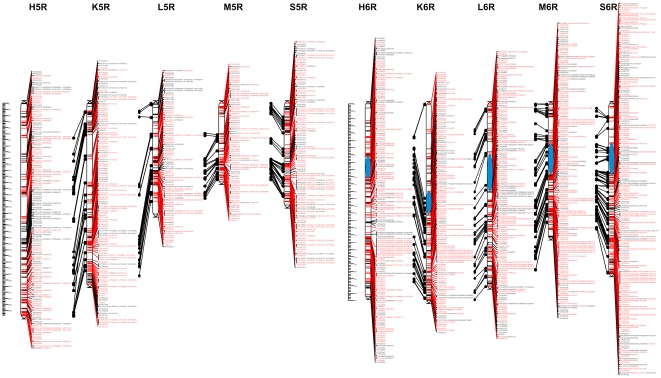
Consensus maps of chromosomes 5R and 6R. Shared and unique markers are shown in red and black, respectively. Common loci are joined by black lines. The chromosomes are oriented with the short arm at the top. The ruler shows the distance in centimorgans (cM) from the top of each chromosome. The approximate centromere locations are shown in blue. For 5R the centromere locations are not shown due to lack of indicative markers.

**Figure 5:**
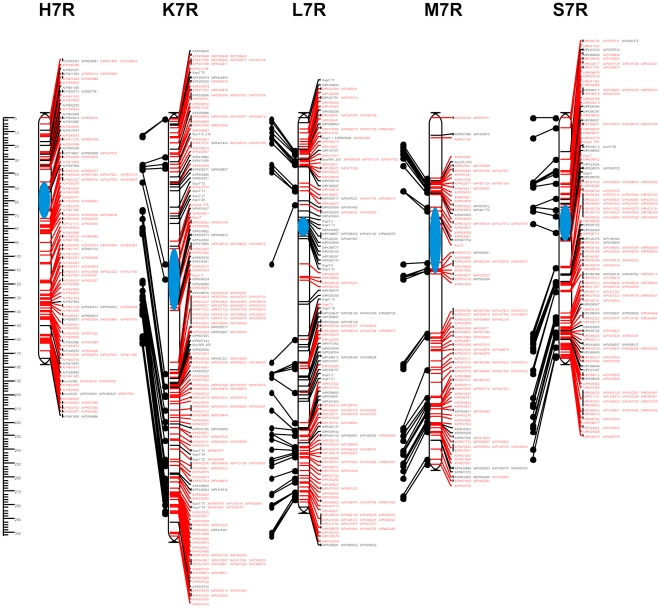
Consensus map of chromosome 7R. Shared and unique markers are shown in red and black, respectively. Common loci are joined by black lines. The chromosome is oriented with the short arm at the top. The ruler shows the distance in centimorgans (cM) from the top of each chromosome. The approximate centromere locations are shown in blue.

After assigning markers to chromosomes and control of monotony, 447 problematic markers causing neighborhood instabilities (on average 12.7 markers per chromosome per population) were identified and removed. As a consequence of removing these markers, a proportion of the remaining markers became no longer linked at the adopted threshold recombination fraction value. These markers were also excluded from subsequent analyses. The markers not assigned to any linkage group at the recombination fraction value of 0.2 constituted 3.9% of the 8303 markers entered into Multipoint Consensus 2.2 (Table 4, Table S3). In total, 7531 marker segregations were used for the construction of consensus maps. This number included 2058 shared markers, with 34 markers segregating in all 5 populations (Table 5). As a result of recalculations of the individual genetic maps, a consensus was achieved, i.e. a consistent order of markers on a given chromosome in all 5 populations. The obtained maps with the changed, consistent order of markers were highly similar to the initial maps of the individual populations. The total length of the consensus map, based on the average length of the chromosome component maps was 1593.0 cM, with an average density of 1.1 cM (Table 6). Graphical representations of the consensus map are shown in Figures 2, 3, 4, 5. Tables S4, S5, S6, S7, S8, S9, S10 contain detailed data on markers from individual chromosomes, from 1R to 7R.

**Table 5:** Summary of mapped markers including shared and unique markers.

Chromosome	Shared markers observed in n populations				All shared markers	Main shared markers	Unique markers	DArT markers	All markers
	n = 2	n = 3	n = 4	n = 5					
1R	199	61	12	3	275	200	243	495	518
2R	139	79	24	0	242	173	247	469	489
3R	124	76	44	10	254	170	248	488	502
4R	301	139	41	1	482	291	323	788	805
5R	151	68	17	1	237	146	317	536	554
6R	162	89	51	16	318	194	408	709	726
7R	149	67	31	3	250	150	204	423	454
Total	1225	579	220	34	2058	1324	1990	3908	4048

**Table 6:** Characteristics of component maps after consensus analysis.

Population and chromosome	Number of markers used for consensus mapping	Map length [cM]	Mean distance between markers [cM]
Chromosome 1R			
H1R	173	208.7	1.2
K1R	205	282.4	1.4
L1R	178	204.9	1.2
M1R	146	182.6	1.3
S1R	229	198.9	0.9
Mean		215.5	1.2
Chromosome 2R			
H2R	241	221.6	0.9
K2R	112	210.2	1.9
L2R	216	226.1	1.1
M2R	188	218.8	1.2
S2R	156	122.7	0.8
Mean		199.9	1.2
Chromosome 3R			
H3R	219	259.2	1.2
K3R	194	238.1	1.2
L3R	179	232.0	1.3
M3R	172	175.4	1.0
S3R	262	221.4	0.9
Mean		225.2	1.1
Chromosome 4R			
H4R	331	307.7	0.9
K4R	307	275.4	0.9
L4R	258	216.8	0.8
M4R	309	216.6	0.7
S4R	358	240.7	0.7
Mean		251.4	0.8
Chromosome 5R			
H5R	224	348.7	1.6
K5R	212	296.7	1.4
L5R	135	181.2	1.3
M5R	117	127.9	1.1
S5R	212	167.2	0.8
Mean		224.3	1.2
Chromosome 6R			
H6R	270	264.2	1.0
K6R	211	270.1	1.3
L6R	249	250.4	1.0
M6R	257	182.9	0.7
S6R	353	233.5	0.7
Mean		240.2	0.9
Chromosome 7R			
H7R	140	173.9	1.2
K7R	224	302.5	1.4
L7R	176	281.4	1.6
M7R	129	250.5	1.9
S7R	189	174.0	0.9
Mean		236.5	1.4
**Total** **Mean**	**7531** **215**	**1593.0** **227.5**	**-** **1.1**

#### Chromosome 1R

Altogether, 931 marker segregations were used to create the chromosome 1R consensus map. A total of 275 markers segregated in more than one population, 199 of these were in common for two populations, and only 3 markers (XrPt400138, XrPt505839 and XrPt506506) were mapped in all 5 populations (Table 5, Figure 2, Table S4). The highest number of markers (229) was placed on the 1R map in population S, and the lowest (146), in population M (Table 4). The average map length was 215.5 cM, with a mean distance between loci of 1.2 cM (Table 6). Markers were not evenly distributed along the chromosome, with marker clustering apparent in certain regions. Three gaps, with distances between neighboring markers larger than 20 cM, were also present (1RS, populations H, K and S).

#### Chromosome 2R

The number of markers placed on the 2R consensus map (Figure 2) ranged from 112 (K) to 241 (H), with the total number of segregations used for consensus mapping of this chromosome equal to 913 (Table 6). Of the 242 shared markers, 139 segregated in two populations and none was common to all 5 maps (Table 5). The lengths of the 2R maps were similar for H, K, L and M, and ranged from 210 to 226 cM. In the case of population S, the map was shorter by almost half (Table 4, Table S5), but at the same time it was the densest of the five maps. One large gap (20 to 34 cM, depending on the population) was observed in the middle of the long arm of the 2R maps of the four remaining populations. The mean distance between loci was 1.2 cM.

#### Chromosome 3R

In total, 1026 segregations were used for consensus mapping of chromosome 3R. This number included 254 shared markers with 10 of these segregating in all populations (Table 3, Table S6). The smallest number of markers was placed on the 3R maps of populations M (172) and L (179), and the largest (262), in population S (Table 6). The linkage map lengths exceeded 200 cM and ranged from 221.4 (S) to 259.2 (H), with the exception of population M, where the map spanned 175.4 cM. The average density of the individual maps was 1.1 cM, and only in the case of the population S was the mean distance between loci below 1 cM. Gaps larger than 20 cM were observed on the long chromosome arm in four populations: K, L, M and S (Figure 3, Table S6). It was found that in spite of the consensus analysis, several markers were not mapped to corresponding locations on the component maps. Such a situation occurred when shared markers co-segregated in one population, while in another population they occupied different map positions. One example of this was marker XrPt509013, which co-localized with markers XrPt402217, XrPt347125 and XrPt347301 in the component map of population K, whereas all four markers were mapped to different locations in the H population.

#### Chromosome 4R

Consensus mapping of chromosome 4R involved the highest number of segregations (1563) and also the highest number of shared markers (482), with 301 and 139 markers being in common for two and three populations, respectively (Table 5). Interestingly, among this large number of markers, only one (XrPt506073) segregated in all populations. The number of markers in the component maps exceeded 300 and ranged from 307 (K) to 358 (S), with the exception of population L, where the genetic map contained 258 loci. All of the component maps spanned over 200 cM, with the H map exceeding 300 cM (Table 6). Markers were distributed very evenly and only one gap was observed, in the distal region of the long chromosome arm, in the case of population K (Figure 3, Table S7). The average interval length (0.8 cM) was the lowest among the seven chromosomes. Similarly to chromosome 3R, there were several inconsistencies in the placement of markers on genetic maps of different populations, e.g. markers Xscsz728L950 and XrPt401071 co-localized on the S map, while in the M map they were separated by approximately 30 cM.

#### Chromosome 5R

The total number of segregations used for consensus mapping of chromosome 5R was 900. Of the 237 shared markers, the majority were in common for 2 or 3 populations (151 and 68, respectively) with one marker (XrPt505721) segregating in all populations (Table 5). The individual maps varied noticeably in length. The longest was that of the population H (348.7 cM), while for population M the map spanned only 127.9 cM (Table 6). The mean distance between loci was 1.2 cM and ranged from 0.8 cM (S) to 1.6 cM (H). However, the distribution of markers was not uniform. Clustering of markers and a higher number of gaps than on the other chromosomes were observed. Two large gaps (over 30 cM) were found in corresponding positions of the 5RS maps in populations H and K. Moreover, a gap in 5RS was apparent in the case of population M (Figure 4, Table S8). A discrepancy in the map location of marker XrPt349332 was observed: in population H it was placed at the end of the long arm, while in the K map it was also located on the long arm, but closer to the middle of the chromosome.

#### Chromosome 6R

For the construction of the 6R consensus map, 1340 marker segregations were used. Among the 318 shared markers (Table 5), 16 were segregating in all populations (Table S9) – the highest number observed in this study. The number of markers placed in component maps ranged from 211 (K) to 353 (S) (Table 4). The average map span was 240.2 cM (from 182.9 cM for population M to 270.1 cM for population K), with a high average map density (0.9 cM). In the case of the component maps, this value ranged from 0.7 cM (M and S) to 1.3 cM (K).The distribution of markers along the 6R maps was rather uniform. Only two gaps were observed on the short arm in the map of population K (Figure 4, Table S9).

#### Chromosome 7R

The 7R consensus map was built using the lowest number of segregations (858). The number of unique segregations was also the lowest (204). On the other hand, the number of shared markers was moderate (250) and included 3 markers (XrPt390749, XrPt402327, XrPt400252) in common for all populations (Table 5). Component maps contained between 129 (M) and 224 (K) markers and spanned 236.5 cM on average (from approximately 174.0 cM for populations H and M, to 302.5 cM for population K), which is comparable with the average map lengths of the other chromosomes (Table 6). Consequently, the mean distance between loci (1.4 cM) was the highest in the case of 7R. Distribution of markers along the 7R genetic maps was not uniform. Marker clusters, as well as four large (one in the 7RL maps of K and M, and two in the 7RS map of population M) and several small gaps (in the case of populations L and S) were apparent (Figure 5, Table S10).

### Segregation distortion

Of the 7531 segregations included in the consensus map, deviation from the expected ratio (p<0.01) was observed for 985 (13.1%). For the component maps, the proportion of distorted markers varied from 3.5% (L) to 33.0% (H), whereas for individual chromosomes these values ranged from 0.6% for 3R in population L to 75.7% for 7R in population H (Table 7). The pattern of distribution of distorted markers among individual chromosomes in the component maps was not uniform. For example, in population H, the second highest percentage of distorted markers was observed in the 1R map, whereas in population S, the percentage of skewed segregations was the lowest for 1R. Similarly, in population L, the highest percentage of distorted markers was observed for 6R, while in populations H and K, the 6R maps were characterized by the lowest percentage of skewed markers.

**Table 7:** Percentage of distorted markers (p<0.01) in component maps.

Chromosome	H	K	L	M	S
1R	46.2	12.2	1.7	5.5	0.9
2R	34.9	17.0	2.3	0.5	10.9
3R	21.0	6.7	0.6	7.0	1.1
4R	31.4	14.7	0.8	25.9	8.9
5R	35.7	14.2	4.4	0.9	2.8
6R	10.0	6.6	9.6	9.3	5.1
7R	75.7	12.1	4.5	21.7	2.1
total	33.0	11.8	3.5	11.7	4.7

Markers with segregation distortion at the 1% level are indicated in Tables S4, S5, S6, S7, S8, S9, S10 by an asterisk. In general, skewed markers were not distributed evenly along the chromosome length; on the contrary, chromosome regions with a high number of distorted markers were easily recognized.

### Integrated map

The integrated map based on data from five component maps consisted of 4048 markers, with the number of markers per chromosome varying from 454 for 7R to 805 for 4R. Unique markers, which segregated in a single population, constituted almost half of the mapped loci (1990), with the number per chromosome ranging from 204 (7R) to 408 (6R). The integrated map comprised 2058 markers (the main shared markers) segregating in more than one population, which corresponded to 1324 unique map locations. Their number varied from 146 for chromosome 5R to 291 for chromosome 4R. Graphical representations of the integrated maps for the individual chromosomes are shown in Figures S1, S2, S3, S4, S5, S6, S7, where the main shared markers and the unique markers are shown in brown and gray, respectively. Lists of all shared and unique markers located on the integrated maps of individual chromosomes are given in Tables S4, S5, S6, S7, S8, S9, S10.

### Comparisons of the component maps and the integrated map (marker number and map lengths)

**Figure 6:**
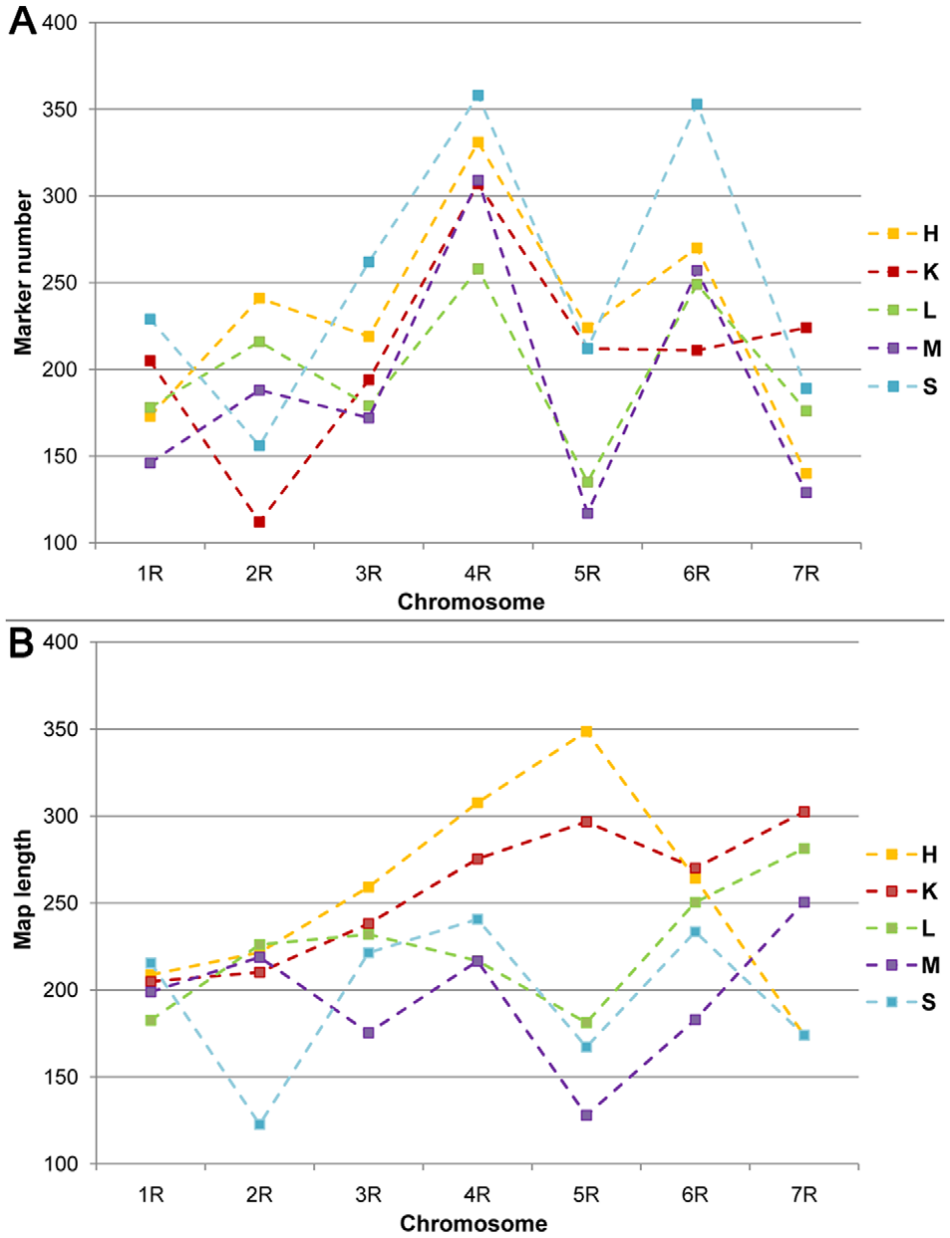
Comparisons of the component maps. A. Marker number per chromosome in individual populations. B. Chromosome map lengths in individual populations.

A comparison of the number of markers placed on individual chromosomes and the chromosome map lengths (Figure 6, Table 8) showed that, while marker numbers were moderately or even highly correlated for the majority of the population pairs, with correlation coefficient values of above 0.7 (and even reaching up to 0.94 for the population pair L and M), there were also cases of very poor correlation (e.g. population pair K and L, with a correlation coefficient value of 0.27). Map lengths were generally not correlated between populations, with the exception of populations L and M, where the correlation coefficient value was 0.63. Similarly, a lack of correlation (correlation coefficient of 0.37) was observed between the total number of markers placed on the individual chromosomes of the integrated map constructed in this study and the physical rye chromosome lengths reported by Schlegel et al. [42].

**Table 8:** Correlation coefficient values for marker number per chromosome (upper values) and chromosome map length (lower values) of the component maps.

	K	L	M	S
H	0.37	*0.72	***0.87	*0.71
	0.35	−0.53	**−0.77	0.22
K		0.27	0.47	*0.70
		0.35	−0.09	0.10
L			***0.94	*0.67
			0.63	−0.01
M				**0.82
				−0.09

*, ** and *** indicate p<0.1, p<0.05 and p<0.01, respectively.

## Discussion

The basic prerequisites for linkage map construction are (i) a suitable mapping population, (ii) a technology generating a sufficient number of markers, and (iii) powerful mapping software.

### Mapping populations

So far, rye linkage maps have been predominantly constructed based on F_2_ populations [3], [5], [7]–[9], [11], [14], [32], with the exceptions of, e.g. the maps created by Bolibok-Bragoszewska et al. [25], and Hackauf et al. [15], where RILs and a BC_1_ population were used, respectively.

Self-incompatibility and severe inbreeding depression, combined with the lack of an efficient methodology for DH production, have hampered the development of mapping populations in rye. In the present study, RIL mapping populations were utilized. Due to the high level of homozygosity in these populations, they may be propagated, thus offering the possibility of repeated sampling in different vegetation periods and at multiple locations. However, the number of genotypes in individual populations was slightly lower than theoretically required to achieve precise mapping which is about 200 individuals [43]. It is known that the use of a population with an insufficient number of genotypes results in incorrect ordering of loci and fragmentation of the linkage groups [43]. On the other hand, the crucial advantage of using RILs lies in the multiple rounds of meiosis that occur before homozygosity is achieved, which gives a greater probability of recombination between closely linked genes and increases the power of testing differences between genotypic classes [44], [45].

The low values of genetic similarity coefficients observed for parental pairs and all possible pairs of lines used in this study demonstrated that they represent a large part of rye genomic diversity. This confirms that the choice of populations was appropriate and provides a high probability of finding polymorphic markers for any given chromosome region. As a result, the integrated map has good genome coverage. Clear differences in the genome structure of the individual populations, that are beneficial for the construction of an integrated map, were revealed by the values of the correlation coefficients obtained for the number of markers per chromosome and chromosome map lengths, and especially by the lack of correlation for the chromosome map lengths. On the other hand, some common genetic basis between populations is necessary for effective bridging of component maps [28], which is based on markers co-segregating in more than one population. From this point of view, the inclusion of two populations with a common parent (K and S) facilitated consensus mapping. This is demonstrated by the fact that the highest number of common DArT markers was observed for this population pair (Table 3).

### Marker choice

DArT markers that detect polymorphisms mainly due to single base-pair changes (SNPs) at restriction enzyme recognition sites, were the predominant marker type used for map construction in this study. SNP polymorphisms account for ∼90% of genetic variation in any organism and are uniformly distributed throughout a genome [22]. The frequency of SNPs in the rye transcriptome is estimated to be 1 SNP per 52–58 bp [46]–[47]. However, due to the dearth of sequence information available for rye, SNP-specific detection assays were limited to 12 Cleaved Amplified Polymorphic Sequence (CAPS) markers [46].

A major advantage of DArT technology is the possibility of genotyping thousands of markers in a fast and cost-effective manner without relying on sequence information. Furthermore, DArT is currently the only accessible method of generating polymorphic and sequence-specific markers in sufficient numbers to enable the construction of a high-density genetic map of rye. The number of SSR markers, which have been widely applied for this purpose in, e.g. barley [48] and maize [49], is limited to ∼400 in rye [10], [12], [20], [50], and other SNP genotyping platforms are still not available for this crop.

The construction of a consensus map is not possible without common markers representing each chromosome. Genotyping of all populations using the same genotyping array, consisting of 11,520 clones, ensured the identification of a sufficient number of markers segregating in more than one population and facilitated the construction of maps with a consistent locus order on a given chromosome. In this study, DArT markers constituted 99.9% of shared markers, whereas only 25 PCR-based markers segregated in more than one population. However, the non-DArT markers, previously mapped in RIL population H and F_2_ progenies of K and L crosses [14], [25], were helpful in assigning linkage groups to individual rye chromosomes and establishing chromosome arm orientation.

### Mapping procedure

Consensus map construction using the Multipoint Consensus 2.2 software package relies on creating a correct order of shared markers within a linkage group. In the opinion of the program creators, the optimal locus order is of greater importance than the establishment of estimated distances between markers [40], and this is clearly visible when map construction is the starting point for map-based cloning efforts. The algorithms implemented in Multipoint Consensus 2.2 [40] were applied here to optimize the map orders during the construction of the presented maps.

To achieve a correct outcome of linkage group construction, high quality genotyping data and mapping algorithms ensuring a suitable stability of resulting map are required. The optimization of a multilocus map requires the resolution of complications originating from high sampling variation of recombination rates, missing data, scoring errors and non-monotonic changes in recombination [40]. Solving the problems resulting from the quality of segregation data is not trivial, especially when the number of markers with significantly skewed segregations is high. In the case of the consensus map presented here, severely distorted segregations (p<0.001) were excluded from data sets. An exception in terms of the threshold level (p<0.0005 instead of p<0.001) was made in the case of population H, since distorted markers were not removed during the construction of the previously published map of this cross: the first DArT-based map of rye [25]. Moreover, the level of segregation distortion was generally higher in this population than in the four other populations utilized in this study (Table 7) [25]. Nevertheless, the excluded skewed markers constituted of only about 7% of the total number of markers. Similar to the present study, severely distorted markers (p<0.001) were excluded prior to linkage analyses in triticale by Alheit et al. [28].

Further problems may be caused by markers interfering with map stability by deviation from the expected increase in recombination rates between a marker and its immediate neighbors. To identify such markers, the ‘control of monotony’ function was used. In the present study, these markers constituted only a small fraction of the data set and were excluded from subsequent analyses.

### Locus order and marker distribution

In most cases, the positions of non-DArT anchor markers on the consensus map were in good agreement with their locations on the respective source maps (F_2_ or RIL). The order of shared markers was also generally consistent between component maps. Minor inconsistencies in map positions were restricted to the same chromosome arms. Situations where a marker was mapped to different chromosome arms in separate component maps were very rare. Minor discrepancies in marker locations are not unusual in consensus maps [28], [51]–[52], and were also observed in the previously published consensus map of rye [19]. Such discrepancies could reflect real differences in genome organization, but they may also be caused by a dependency of the estimated gene orders on sample size or by differences in local recombination frequencies between populations [28], [51].

Seventy-two DArT markers (1.8%) were found on multiple chromosomes of different populations. Because DArT is a hybridization-based assay, these markers effectively identify multiple genomic regions sharing sequence homology and the polymorphic region can be different in separate crosses [21]. Multicopy DArT markers were observed with a similar frequency in triticale [28], sorghum [51] and barley [52] (1.8%, 1.4% and 1.8%, respectively).

Despite utilizing several methods of map optimization, regions containing recombination gaps were observed in the constructed map, mostly in the distal regions of the chromosomes. The locations of recombination gaps were remarkably similar in the separate populations for all chromosomes except 4R. The previously published rye consensus map [19], based on five F_2_ populations, which included 501 loci of various types (e.g. RFLP, RAPD, SSR), also contained recombination gaps in the terminal parts of chromosomes 1RS, 1RL, 3RS, 4RL, 5RL and 6RS. In general, the occurrence of recombination gaps is a common feature of all available rye maps, including the densest published so far, an AFLP-based map produced by Bednarek et al. [11], and the DArT-based map of Bolibok-Brągoszewska et al. [25]. Unfortunately, the precise comparison of gap locations in different rye populations is not possible in many cases due to the low number of common markers. The existence of recombination gaps in similar locations in the component rye maps presented here could be the result of DArT marker limitations in detecting polymorphism in certain genome regions. However, at least some of the gaps (e.g. those present on the short arm of 1R and 6R) are located in the same regions as gaps identified in the consensus map of Gustafson et al. [19], which was constructed using other types of markers. This suggests that rather than indicating a DArT-specific limitation, these gaps are actually conserved in the rye genome and reflect regions with a higher than average frequency of recombination (recombination hot spots). Alternatively, these recombination gaps may represent genome fractions with similar ancestry, as proposed by Mace et al. [51] and van Os et al. [53], who observed large recombination gaps in a consensus map of sorghum and an ultra-dense map of potato, respectively.

### Segregation distortion

Segregation distortion is a common phenomenon in rye [5], [7]–[8], [15]–[17] and other plants such as triticale [27]–[28], maize [49], sorghum [51] and potato [53]. Because different stringency levels were applied for the removal of severely distorted markers prior to linkage mapping in this study (for the reasons outlined above), it is not possible to directly compare the proportion of skewed markers present in the component maps between population H and the other 4 populations. Nevertheless, it was noticeable that individual chromosomes within a component map varied in the proportion of distorted markers they contain (e.g. from 0.5% for 2R to 25.9% for 4R in the case of the component maps for population M). Moreover, the chromosomes with the highest or the lowest percentage of distorted markers were different in the separate populations. Large differences in the percentage of distorted markers present on individual chromosomes (from 0 to 100%) were also observed in triticale by Alheit et al.[28], who attributed this to the different ways of producing the individual mapping populations used in their study (five DH populations and one F_2_ population). All the populations employed in the present study were RILs and all the component maps were produced using the same methods and mostly with the same marker type. Therefore, the differences in the distribution of distorted markers may be attributed to (i) the different allelic composition of the parents of the individual component populations in the respective chromosomal regions, i.e. alleles with a more or less equal influence on survival rate in both parents vs. alleles with a stronger negative or positive influence on the survival rate in one of the parents, and (ii) to differences in the number of individuals between the separate mapping populations. The latter explanation is especially relevant for population H, which was the smallest population used in this study and, hence, the most likely to be characterized by a non-random representation of alleles.

### Integrated map

The integrated rye map reported here, containing 4048 loci (3908 DArTs), represents the largest collection of molecular markers currently available for rye genome analyses. Due to the use of multiple mapping populations, a more than two-fold increase in the number of genetically mapped markers was achieved in comparison with the first DArT-based map of rye [25]. Moreover, in comparison with the previously published rye consensus map [19], our integrated rye map comprises 8-times more loci. In the present study, we employed sequence-specific, transferable DArT markers, assayed in a largely automated manner using a microarray-based technology and that were easily accessible through a genotyping service. Therefore, the presented map constitutes a valuable resource for rye and triticale geneticists and breeders, and is a significant step forward for rye genomics.

One interesting feature of the constructed integrated map is the lack of correlation between the number of markers and the physical length of the rye chromosomes. This phenomenon is consistent with the strategy used to generate rye DArT markers. The genome complexity reduction method used for the development of the rye genotyping panel and for the genotyping assay, involved digestion with the restriction endonuclease *Pst*I. This enzyme is CpNpG methylation-sensitive and therefore is often used to target single- and low-copy DNA/transcriptionally or biologically active euchromatic DNA, since most repetitive sequences are completely methylated at this site [54]–[55]. Such an approach is especially well suited for analyzing the rye genome because of its very high proportion of repetitive sequences: 92% [20]. Previously, *Pst*I was used in rye research to create single- and low-copy genomic DNA libraries for the development of SSR markers [50]. In cucumber, a higher correlation was observed between the number of markers and euchromatic chromosome length than between marker number and pachytene chromosome length for a map constructed with SSR markers derived from non-repetitive genome sequences [56]. Unfortunately, to our knowledge, there are no published reports describing euchromatic chromosome length in rye. Nevertheless, cytogenetic observations have shown that euchromatin is not proportionally distributed among the chromosomes of rye, as four (1R, 2R, 3R and 7R) have large blocks of heterochromatin at the telomeres of both arms, while the remaining three chromosomes (4R, 5R, 6R) have heterochromatic blocks at the telomeres of the short arms. In addition, blocks of interstitial heterochromatin are present on every chromosome [44], [57].

### Potential applications

The presented maps are suitable for exploitation in a range of genomic, biotechnological and breeding applications. The very high density map may serve as a reference in rye linkage mapping, facilitating the construction of genetic maps for newly developed populations. The map could also accelerate association mapping in rye by facilitating the estimation of linkage disequilibrium, as well as the detection of QTLs via traditional interval mapping. The high map saturation will be highly advantageous during BAC clone anchoring based on the use of DArT arrays, as described for wheat by Paux et al. [58]. Our results are also likely to accelerate research on triticale, an intergeneric hybrid between wheat and rye. The usefulness of DArT-based rye genomic resources for analyses of this crop was recently demonstrated by Badea et al. [26], Tyrka et al. [27] and Alheit et al. [28]. The unique value of the presented integrated map would significantly increase once the sequencing of DArT clones from the rye genotyping panel is completed [http://www.diversityarrays.com/faq.html#n67]. Nevertheless, *in situ* hybridization experiments involving mapped DArT clones are advisable in order to align certain map features with the physical organization of rye chromosomes.

### Conclusion

A highly saturated integrated map of rye containing over 4000 loci and a consensus map with a highly consistent locus order, constructed using a suitable marker type, mapping populations and a software package implementing powerful algorithms for map order optimization, represent valuable resources for rye and triticale genomics and breeding, and are an excellent starting point for more in-depth studies on rye genome organization.

## Supporting Information

Figure S1 **Integrated linkage map of rye chromosome 1R. The main shared and unique markers are shown in brown and gray, respectively.**
(TIF)

Figure S2 **Integrated linkage map of rye chromosome 2R. The main shared and unique markers are shown in brown and gray, respectively.**
(TIF)

Figure S3 **Integrated linkage map of rye chromosome 3R. The main shared and unique markers are shown in brown and gray, respectively.**
(TIF)

Figure S4 **Integrated linkage map of rye chromosome 4R. The main shared and unique markers are shown in brown and gray, respectively.**
(TIF)

Figure S5 **Integrated linkage map of rye chromosome 5R. The main shared and unique markers are shown in brown and gray, respectively.**
(TIF)

Figure S6 **Integrated linkage map of rye chromosome 6R. The main shared and unique markers are shown in brown and gray, respectively.**
(TIF)

Figure S7 **Integrated linkage map of rye chromosome 7R. The main shared and unique markers are shown in brown and gray, respectively.**
(TIF)

Table S1 **List of PCR-based markers used for genotyping.**
(XLS)

Table S2 **DArT marker segregations for five RIL populations**
(XLS)

Table S3 **Lists of markers excluded during map construction.**
(XLS)

Table S4 **Consensus map of chromosome 1R and orders of shared and unique markers within the 1R integrated map.**
(XLS)

Table S5 **Consensus map of chromosome 2R and orders of shared and unique markers within the 2R integrated map.**
(XLS)

Table S6 **Consensus map of chromosome 3R and orders of shared and unique markers within the 3R integrated map.**
(XLS)

Table S7 **Consensus map of chromosome 4R and orders of shared and unique markers within the 4R integrated map.**
(XLS)

Table S8 **Consensus map of chromosome 5R and orders of shared and unique markers within the 5R integrated map.**
(XLS)

Table S9 **Consensus map of chromosome 6R and orders of shared and unique markers within the 6R integrated map.**
(XLS)

Table S10 **Consensus map of chromosome 7R and orders of shared and unique markers within the 7R integrated map.**
(XLS)
